# Leveraged Vaccination to Alleviate Original Antigenic Sin for Enhancing Broad‐Neutralizing Antibody Response against SARS‐CoV‐2 Omicron Subvariants

**DOI:** 10.1002/mco2.70273

**Published:** 2025-07-07

**Authors:** Guangxu Zhang, Qian Wang, Kai Ji, Yuanzhou Wang, Wei Xu, Jie Zhou, Zezhong Liu, Ruixue Xiu, Lixiao Xing, Jianghao Zhou, Yuren Shi, Xishan Lu, Xuanyi Wang, Bo Ying, Lu Lu, Shibo Jiang

**Affiliations:** ^1^ Key Laboratory of Medical Molecular Virology (Ministry of Education/National Health Commission/Chinese Academy of Medical Science), Shanghai Institute of Infectious Disease and Biosecurity, School of Basic Medical Sciences Fudan University Shanghai China; ^2^ Suzhou Abogen Biosciences Co., Ltd. Suzhou Jiangsu China; ^3^ Department of Pharmacology & the Key Laboratory of Smart Drug Delivery School of Pharmacy Ministry of Education Fudan University Shanghai China

**Keywords:** original antigenic sin (OAS), Omicron subvariants, mRNA vaccine, broad‐neutralizing antibody (bnAb)

## Abstract

Original antigenic sin (OAS), or immune imprinting, triggered by severe acute respiratory syndrome coronavirus 2 (SARS‐CoV‐2) ancestral (WT) strain vaccine, or infection, has led to weakened neutralizing antibody response against Omicron variant like BA.2 and subvariant like XBB. This calls for the development of an innovative booster vaccine, or vaccination strategy, that will eliminate, or attenuate, OAS, thus, enhancing broad‐neutralizing antibody (bnAb) response. Accordingly, we herein proposed a leveraged vaccination strategy to counter the OAS effect by controlling the antigenic distance of booster vaccine and increasing boost vaccination frequency. We found that prime with WT–RBD and boost with XBB–RBD resulted in significantly higher bnAb response against most Omicron subvariants tested than that after prime with WT–RBD and boost with BA.2–RBD because the antigenic distance between WT–RBD and XBB–RBD is much longer than that between WT–RBD and BA.2–RBD. An additional boost with XBB–RBD further enhanced bnAb response. These findings indicate that a leveraged vaccination approach based on antigenic distance could be effective in reducing OAS, thereby strengthening bnAb response against SARS‐CoV‐2 Omicron subvariants. As such, this vaccination strategy could be just as effective in combating other fast‐evolving RNA viruses known for their high transmissibility and infectivity.

## Graphical abstract

The leveraged vaccination strategy we proposed based on antigenic distance and boost frequence is effective to alleviate original antigen sin (OAS) or immune imprinting from prior exposure of the original SARS‐CoV‐2 antigen. We showed that boost twice with XBB–RBD vaccine having longer antigenic distance than BA.2–RBD is more effective to attenuate OAS than BA.2–RBD, suggesting that this leveraged vaccination strategy is feasible to alleviate OAS and enhance broad‐neutralizing antibody (bnAb) response against Omicron subvariants.

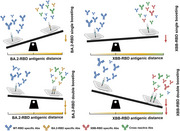

## 1 Introduction

In 2023, the World Health Organization declared that the coronavirus disease (COVID‐19) pandemic was no longer a public health emergency of international concern [1]. Nevertheless, an ever‐increasing number of severe acute respiratory syndrome coronavirus 2 (SARS‐CoV‐2) Omicron subvariants have been cited as variants of concern, and these subvariants continue to emerge and evolve (Figure S1A), posing a threat to public health [2]. For example, the rapid evolution of a single L455S mutation in BA.2.86 resulted in wide global transmission of the JN.1 derivative [3, 4].

To counter this rapidly evolving virus, mRNA booster vaccine candidates based on the antigens from updated strains were developed. However, upon inoculation with the updated vaccine, original antigenic sin (OAS), or immune imprinting, was still triggered by prior vaccination for the SARS‐CoV‐2 ancestral wild‐type (WT) strain [5, 6]. It is well known that the hierarchy of broadly neutralizing antibody (bnAb) response can be altered in individuals with preexisting imprinted responses upon exposure to a novel pandemic virus [7]. For instance, the levels of serum neutralizing antibodies (nAbs) against the pseudotyped WT with D614G mutation (WT–D614G) in the cohort receiving an updated mRNA booster vaccine were notably greater in the groups boosted with both BA.5 bivalent mRNA vaccine [6] and XBB monovalent mRNA vaccine [5, 8].

The “antigenic distance hypothesis” [9], aiming to mitigate OAS effect and improve bnAb response, elucidate that variations in vaccine efficacy is associated with the difference in antigenic distances among vaccine strains [10]. Indeed, repeated exposures can counteract bnAb response to new vaccines or infection types in people, thereby triggering specific antibody responses [11]. However, other studies have suggested that antibody reactions remain largely WT cross‐reactive in most subjects, even after several exposures [6]. Apart from the absence of consensus, a clear understanding of these interactions is confounded by the complexity of serum backgrounds of hybrid immunity from individual cohorts [12] when followed by infection or vaccination. Therefore, both complications call for a systematic approach toward vaccine development with the aim of avoiding the effect of OAS triggered by the receptor‐binding domain (RBD) in spike (S) protein of WT (WT–RBD).

**FIGURE 1:**
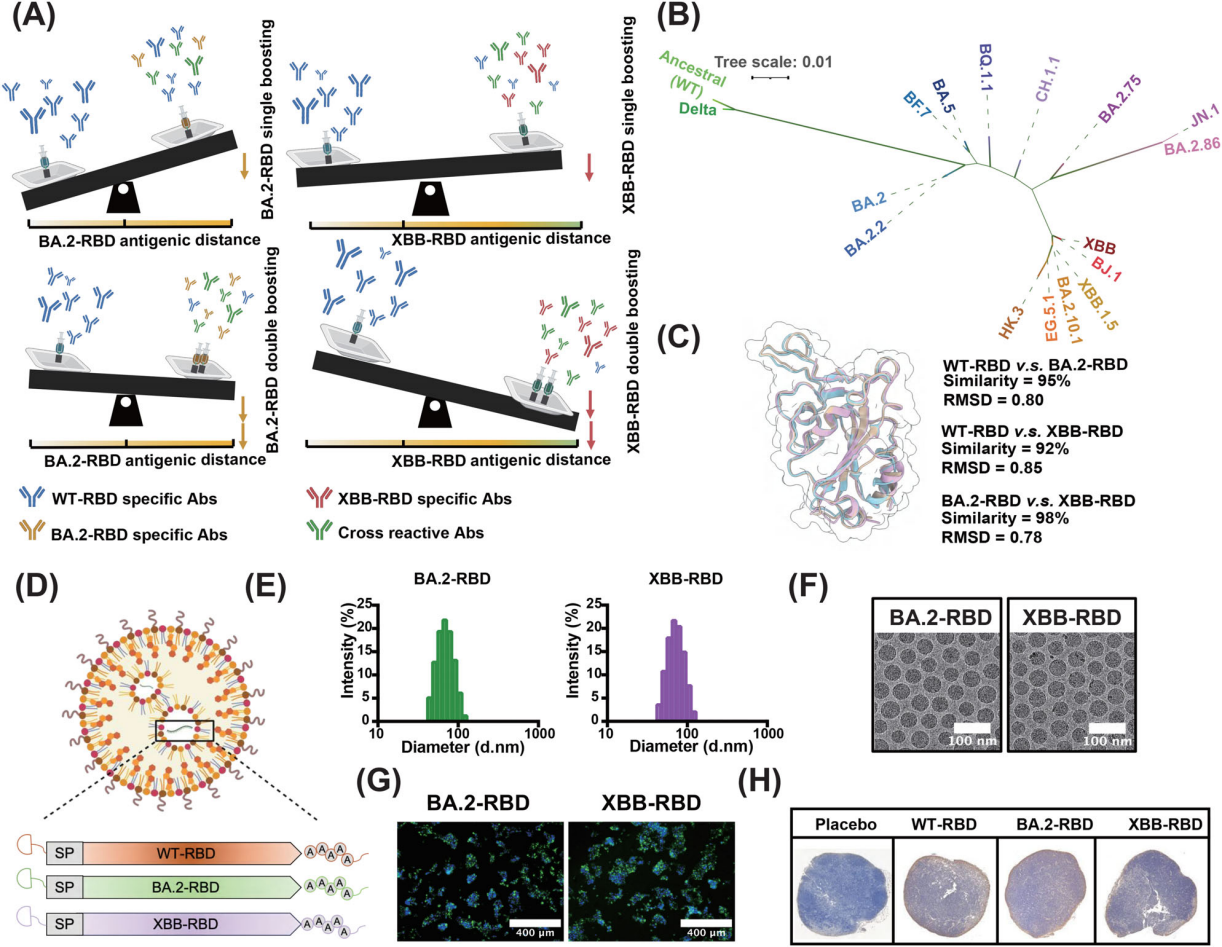
Proposed leveraged vaccination strategy, phylogenetic analysis of receptor binding domain (RBD) of SARS‐CoV‐2 WT and variants, and the construction of RBD mRNA booster vaccines. (A) Proposed leveraged vaccination strategy. (B) Phylogenetic analysis and sequence comparisons among representative RBDs of emerging SARS‐CoV‐2 variants. (C) Structure alignments of the ancestral WT (PDB ID: 7KMG), BA.2 (PDB ID: 7UB5), and XBB (PDB ID: 8IOU) RBDs by root mean square deviation. (D) mRNA based on BA.2–RBD or XBB–RBD was transcribed in vitro and encapsulated into lipid nanoparticles (LNPs). (E) Intensity‐size graph was presented for dynamic light scattering measurement. (F) Cryo‐TEM images of BA.2 and XBB mRNA–LNP in a scale bar of 50 nm. (G) Indirect immunofluorescence showed specific mRNA–LNP expression in vitro. (H) Immunohistochemistry (IHC) was used to evaluate mRNA–LNP expression at the lymph nodes in mice. RBDs were located within the subcapsular and medullary sinus of the draining lymph nodes (LNs).

Therefore, we herein proposed a leveraged vaccination strategy to alleviate OAS from prior exposure of the original antigen. We reasoned that antigenic distance between booster vaccines would be foundational to this strategy and a determinant of bnAb response. In short, this means that we should select an antigen with antigenic distance as long as possible for the development of booster vaccines. To validate our strategy, a phylogenetic analysis was conducted on amino acid sequences of RBD from past epidemic strains, the results of which confirmed the sequence‐based genetic distances. We then investigated this strategy by choosing a booster vaccine with longer antigenic distance and/or by increasing the frequency of antigen boosts (Figure 1A). For a better understanding of the following illustration, we note that monovalent vaccine XBB.1.5 was derived from Omicron variant BA.2 and that they exhibit longer and shorter sequence‐based genetic distances, respectively. Here, we found that the serum nAb titer in mice primed with WT–RBD and boosted twice with XBB–RBD (WT–XBB–XBB) was significantly higher than that in mice primed with WT–RBD and boosted twice with BA.2–RBD (WT–BA.2–BA.2) against the SARS‐CoV‐2 Omicron subvariants tested, suggesting that this leveraged vaccination is able to alleviate OAS, thus enhancing the bnAb response against Omicron subvariants. Therefore, the present research offers a foundational approach based on the antigenic distance hypothesis for future booster vaccine development and immunization strategies against SARS‐CoV‐2 and other fast‐evolving RNA viruses.

## 2 Results

### 2.1 Construction of mRNA Booster Vaccines Expressing RBD of SARS‐CoV‐2 WT Strain, Omicron Variants BA.2, and Subvariant XBB, Respectively

To validate our leveraged vaccination strategy based on antigenic distance, a phylogenetic analysis (Figure 1B) was conducted on amino acid sequences from past epidemic strains of the RBD. This analysis confirmed the sequence‐based genetic distances (Figure S1B). Then, we selected the RBD from the ancestral SARS‐CoV‐2 (WT) strain, one Omicron variant BA.2, and one Omicron subvariant XBB, which exhibit shorter and longer sequence‐based genetic distances, respectively. Structural alignment also revealed that the RBDs of BA.2 and XBB were structurally distinct from that of WT RBD (Figure 1C) since the root mean square distance of the structural alignment was 0.8 between WT and BA.2–RBD and 0.85 between WT and XBB–RBD. These were then used to design mRNA booster vaccines based on WT–RBD, BA.2–RBD, and XBB–RBD (Figure 1D). In our previous studies [13], the mRNA booster vaccine based on the RBD of SARS‐CoV‐2 WT strain, termed as WT–RBD, was validated as effective and approved in Indonesia. Expanding upon this research, we designed and synthesized mRNA constructs for the BA.2–RBD and XBB–RBD. Subsequently, the mRNA constructs were encapsulated into lipid nanoparticles (LNPs) using microfluidic chips. Dynamic light scattering (DLS) analysis showed particle sizes of 70 nm in BA.2–RBD and 67 nm in XBB–RBD (Figure 1E). Cryo‐electron microscopy (Cryo‐EM) revealed a convergent spherical morphology of the nanoparticles (Figure 1F). Furthermore, both in vitro and in vivo transfections demonstrated a broad expression of each mRNA–LNP formulation. Indirect immunostaining demonstrated that proteins expressed from BA.2–RBD and XBB–RBD could be recognized by antibodies targeting SARS‐CoV‐2 (Figure 1G). A positive RBD signal by immunohistochemistry (IHC) was detected in the popliteal lymph nodes in mice (Figure 1H).

### 2.2 XBB–RBD mRNA Booster Vaccine Elicited Strong bnAb Responses against Most Omicron Subvariants Tested

**FIGURE 2:**
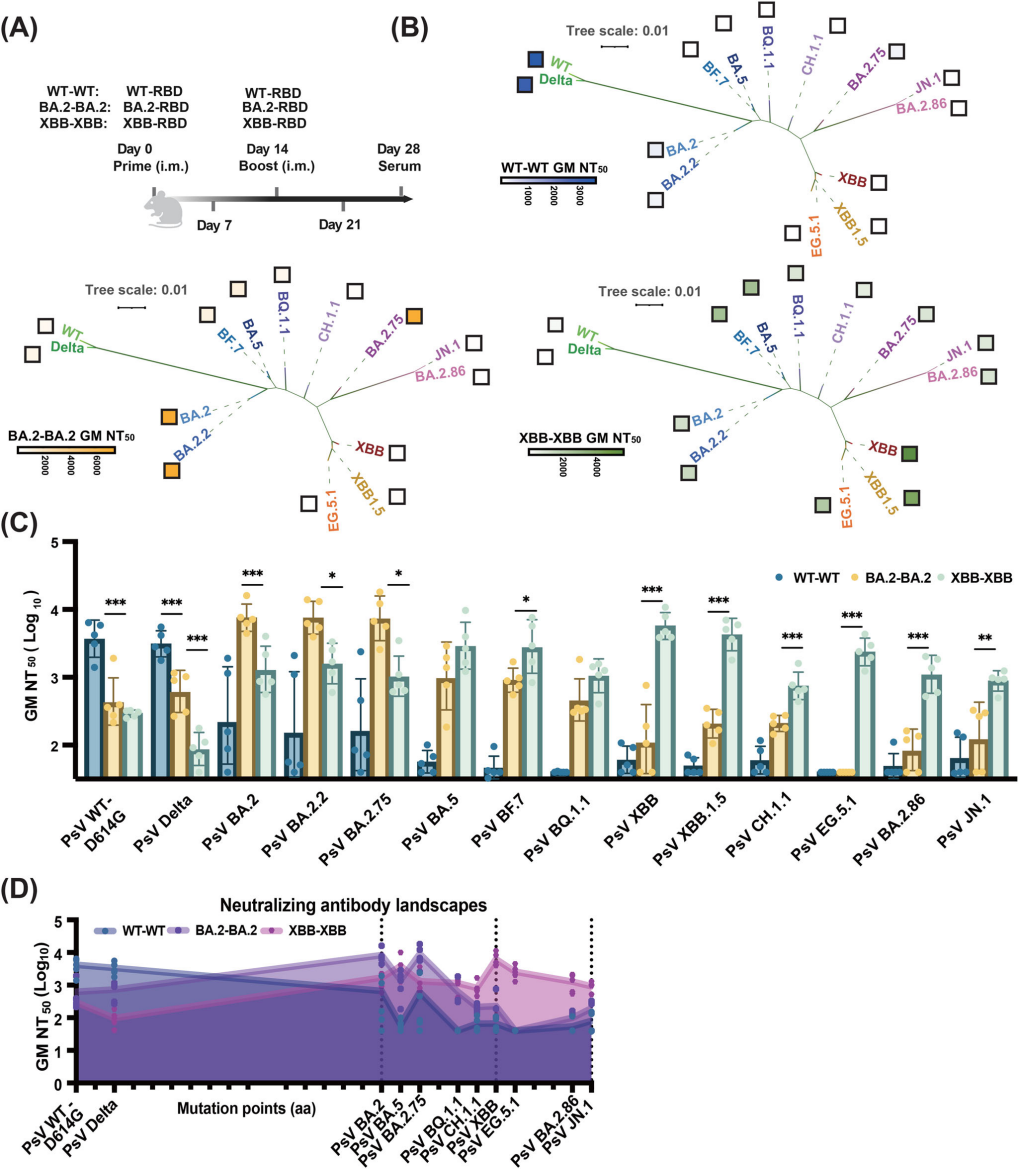
XBB–RBD mRNA booster vaccine elicited broadly neutralizing antibody (bnAb) response against SARS‐CoV‐2 Omicron subvariants. (A) Timeline of vaccination and serum sample collection from BALB/c mice (*n* = 5). (B) Cross‐reactive nAbs against SARS‐CoV‐2 variants superimposed onto a phylogenetic analysis based on differences in RBD sequences. (C) Pseudovirus neutralization titers of sera from mice immunized with two shots of 1 µg of WT–, BA.2–, or XBB–RBD mRNA booster vaccine at day 28 against a panel of 14 SARS‐CoV‐2 variants (*n* = 5). Statistical analyses were performed using one‐way ANOVA for comparison. *p* Values were labeled above, or *p < 0.05, **p < 0.001, ***p < 0.0001. (D) Neutralizing antibody landscapes of sera from mice primed and boosted with 1 µg of WT–RBD, BA.2–RBD, or XBB–RBD, respectively, at day 28 against a panel of 11 SARS‐CoV‐2 variants with distinct mutations in their RBD (Figure S3).

To evaluate the efficacy of these mRNA booster vaccines with genetically distinct RBDs, mice were intramuscularly primed and boosted with homologous RBD vaccine (Figure 2A) of 1 µg each at a 14‐day interval (*n* = 5). The mice were divided into three groups: WT–WT, BA.2–BA.2, and XBB–XBB. Serum samples were collected 28 days after the prime immunization injection (Figure S2A).

We found that WT–WT antisera neutralized potently against WT–D614G and Delta, but weakly against BA.2 lineages. Notably, BA.2–BA.2 antisera effectively neutralized BA.2 lineages (Figure 2B), including BA.2.2 and BA.2.75, with geometric mean (GM) NT_50_ values ranging from 7344 to 7547. However, its neutralization titers decreased approximately 7.4‐fold and more against BA.5 and subsequent lineages (Figures 2C and S2B). In contrast, XBB–XBB antisera exhibited significantly enhanced neutralizing activity against BA.2, BA.5, XBB, EG.5.1, and even the recent BA.2.86 and JN.1 variants, with GM NT_50_ values of 1299, 2899, 5703, 2364, 1099, and 882, respectively (Figure 2C), indicating XBB–RBD, as a vaccine candidate, could induced bnAbs against most of the emerging strain as reported for the XBB.1.5 booster vaccine [14]. Furthermore, the neutralization results between pseudoviruses and authentic viruses were strongly correlated (Figure S2C). Cumulative mutations in the RBD (Figure S3A,B) contributed to the genetic distance among the variants. To visualize this through antigenic cartography, we mapped the neutralizing antibody landscapes of the antisera by evaluating their neutralization activity against 14 SARS‐CoV‐2 variants with distinct mutations in their RBDs. Significantly, six substitutions on the RBD differed their neutralizing antibody landscapes (Figure 2D). We also integrated the neutralizing activity data with phylogenetic analysis to reflect the cross‐reactive nature of the antiserum nAbs, as the WT–RBD, BA.2–RBD, and XBB–RBD mostly neutralizing their own lineages (Figure 2B). Strong nAb responses were observed only against WT and Delta in the WT–WT antisera, BA.2, BA.2.2, and BA.2.75 in the BA.2–BA.2 antisera, and various Omicron subvariants in XBB–XBB antisera. These results indicated that prime and boost with the RBD vaccine from the same strain can induce nAb response against only this strain and its close evolutionary branches.

### 2.3 Prime with WT–RBD and Boost with XBB–RBD Resulted in Weakened OAS and Enhanced bnAb Response against Most Omicron Subvariants Tested Compared with that from Prime with WT–RBD and Boost with BA.2–RBD

**FIGURE 3:**
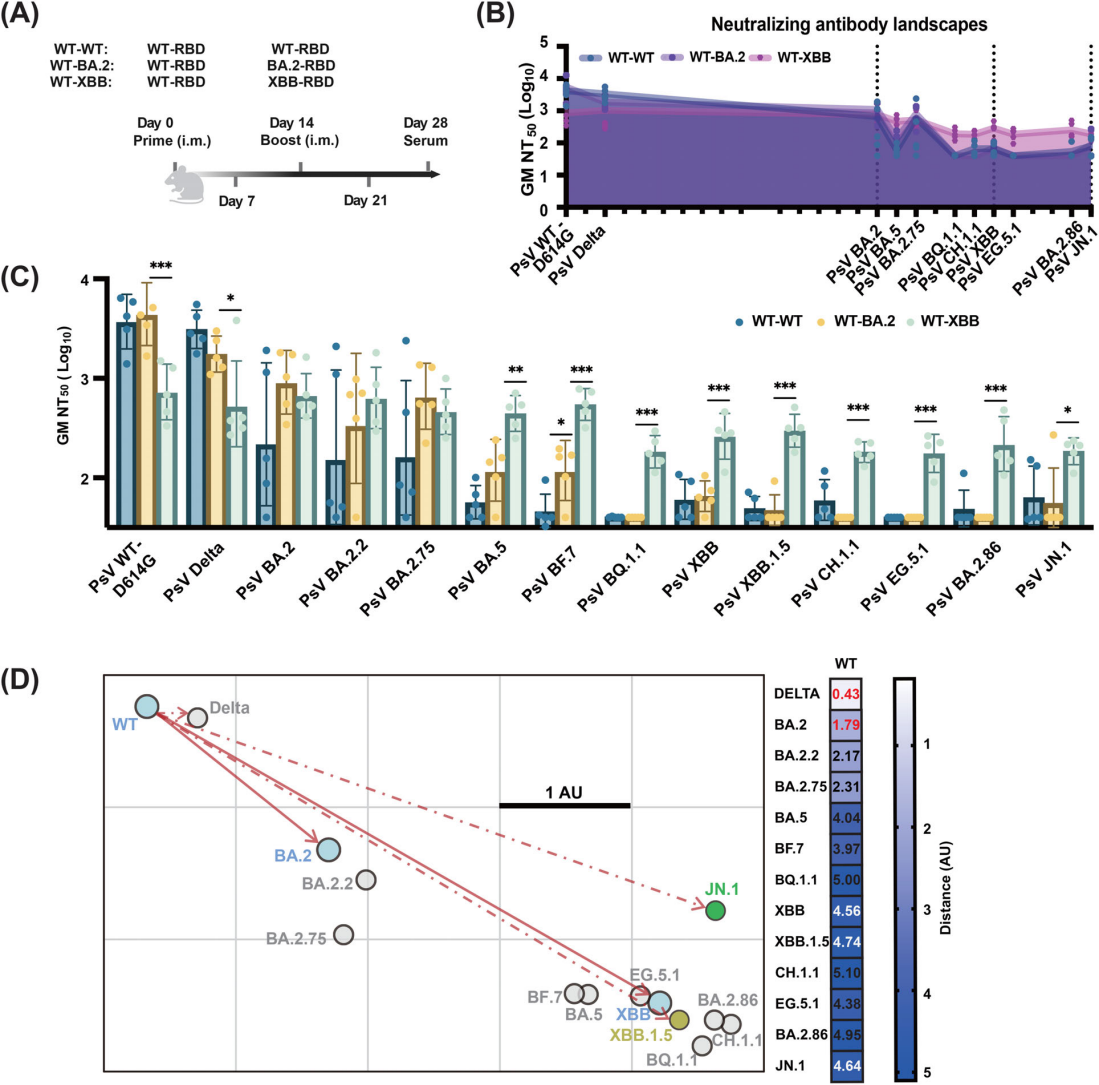
XBB–RBD booster vaccine induced lower original antigenic sin (OAS) after prior exposure to WT–RBD. (A) Timeline of vaccination and serum sample collection from BALB/c mice (*n* = 5). (B) Neutralizing antibody landscapes of sera from mice immunized with WT–WT, WT–BA.2, and WT–XBB at day 28 against a panel of 14 SARS‐CoV‐2 variants with distinct mutations in their RBD (Figure S3). (C) Pseudovirus neutralization titers of sera collected at week 6 from mice primed with 1 µg of WT–RBD and boosted with 1 µg of BA.2–RBD or XBB–RBD against a panel of 14 SARS‐CoV‐2 variants (*n* = 5). All results were statistically performed using one‐way ANOVA. The statistical significance is displayed as follows: *p < 0.05, **p < 0.001, ***p < 0.0001. (D) Antigenic cartography was generated using pseudovirus neutralization data. The length of the square on the grid represents one antigenic unit. Virus positions are shown in closed circles. 1 AU represented approximately twofold change in neutralization.

To assess the impact of boosters with varying antigenic distances on OAS, mice were primed with WT–RBD mRNA booster vaccine and boosted with 1 µg of WT–RBD (WT–WT), BA.2–RBD (WT–BA.2), or XBB–RBD (WT–XBB) mRNA booster vaccine 14 days later (*n* = 5) (Figure 3A).

We found that antisera of the WT–XBB group exhibited strong nAb activity against most Omicron subvariants tested, including BA.2, BA.2.2, BA.2.75, BA.5, BF.7, BQ.1.1, XBB, XBB.1.5, CH.1.1, EG.5.1, BA.2.86, and JN.1 with GM NT_50_ ranging from 175 to 725. Meanwhile, WT‐BA.2 antisera were highly effective against BA.2, BA.2.2, and BA.2.75, less effective against BA.5 and BF.7, and completely ineffective against BQ.1.1, XBB, XBB.1.5, CH.1.1, EG.5.1, BA.2.86, and JN.1 (Figure 3B,C). These findings suggest that a prime with WT–RBD, as prior exposure, followed by a single boosting of XBB–RBD improves bnAb response against Omicron subvariants (Figure 3B,C).

Based on neutralizing activity, we then constructed an antigenic map to visualize antigenic distances. We found that the antigenic distance between WT and BA.2 and between WT and XBB was 1.79 AU and 4.56 AU, respectively, while that between WT and Delta, XBB.1.5, or JN.1 was 0.43, 4.74, or 4.64, respectively (Figure 3D). These data suggest that unlike WT–BA.2 vaccination, WT–XBB vaccination can elicit nAbs to effectively neutralize most Omicron subvariants tested because the antigenic distance between WT and these Omicron subvariants is similar to that between WT and subvariant XBB, while it is much longer than that between WT and variant BA.2, confirming that this leveraged vaccination based on antigenic distance can weaken the effect of OAS and improve the bnAb response against Omicron subvariants.

### 2.4 Double XBB–RBD Boosting Vaccination Further Attenuated WT–RBD‐Derived OAS Effect

**FIGURE 4:**
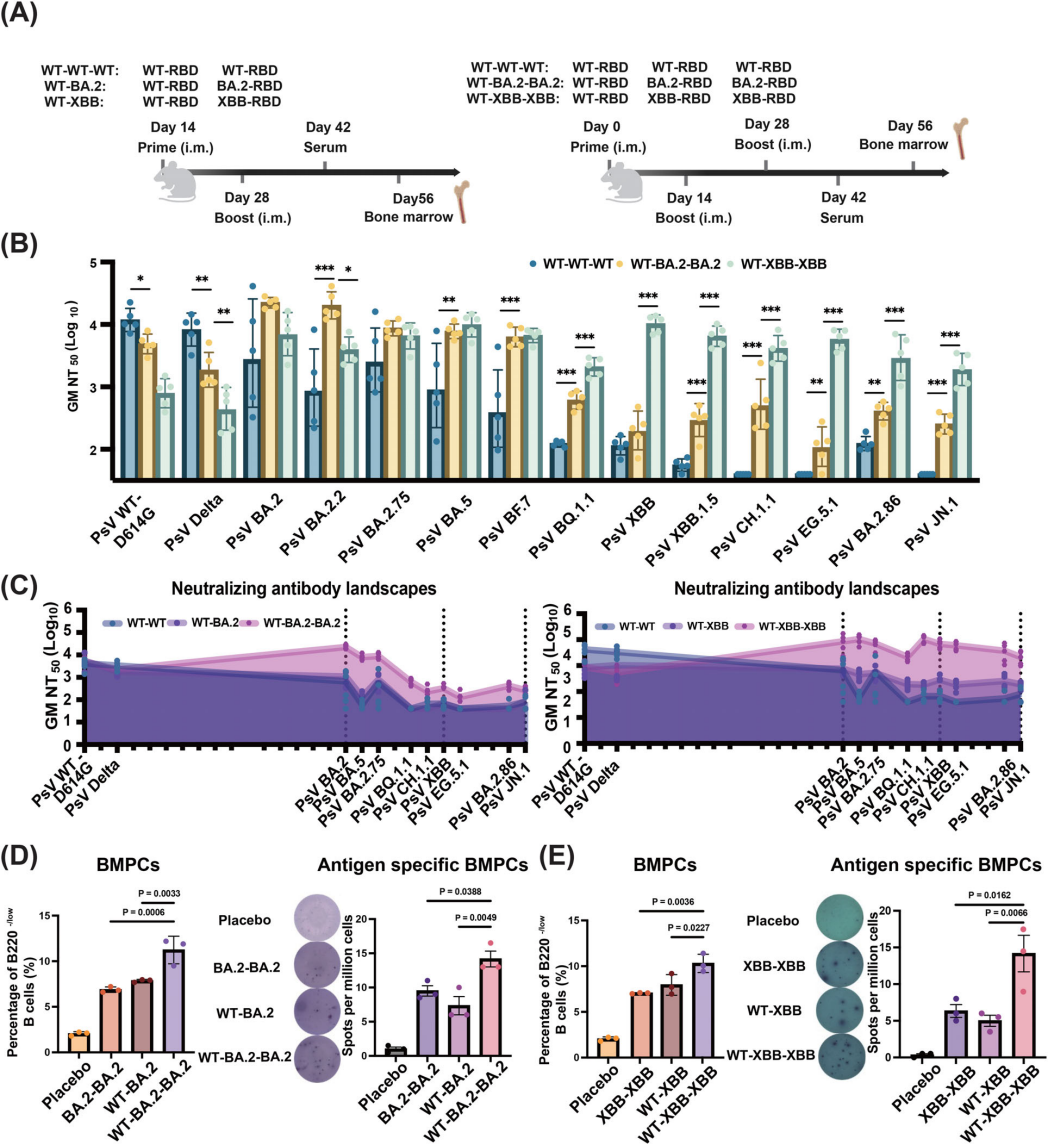
Double XBB–RBD booster vaccination resulted in further improvement of broad nAb responses. (A) Timeline of double booster vaccination and sample collection from BALB/c mice (*n* = 5). (B) Pseudovirus neutralization titers of sera collected from BALB/c mice primed with 1 µg WT–RBD and boosted twice with 1 µg of BA.2–RBD or XBB–RBD against a panel of 14 SARS‐CoV‐2 variants (*n* = 5). All results were statistically performed using one‐way ANOVA. The statistical significance is displayed as follows: *p < 0.05, **p < 0.001, ***p < 0.0001. (C) Neutralizing antibody landscapes of sera from mice boosted once or twice against a panel of 14 SARS‐CoV‐2 variants with a total of 27 mutation points on RBD. (D–E) Bone marrow plasma cells (BMPCs) (B220^−/low^, CD138^+^, CD44^+^) collected at 4 weeks after last immunization were determined by flow cytometry. ELISPOT analysis of RBD‐specific BMPCs was performed. Statistical analyses were performed using one‐way ANOVA for comparison.

To further strengthen leveraged vaccination, we implemented a double XBB–RBD boosting vaccination after prime with WT–XBB (WT–XBB–XBB), and used WT–WT–WT and WT–BA.2–BA.2 as controls (Figure 4A).

As shown in Figure 4B, the GM NT_50_ of WT–XBB–XBB is in the range of 1898–10,210 against most Omicron subvariants tested, significantly higher than that of WT–XBB (GM NT_50_: 175–725) (Figure 3C), suggesting that double XBB–RBD boosting is much more effective than single XBB–RBD in alleviating OAS effect and strengthening bnAb response against Omicron subvariants. However, while the double BA.2–RBD boost could increase nAb response against BA.2 and BA.2.75, it had no effect in boosting immunity against XBB and related subvariants (Figures 3B,C and S4A). These results demonstrate that leveraged vaccination is highly effective in mitigating OAS and improving bnAb responses against evolving Omicron subvariants.

To evaluate the long‐term effects of the leveraged vaccination, we tracked bone marrow‐resident plasma cells (BMPCs) using the gating strategy shown in Figure S5 via flow cytometry. Results showed that the percentage of bone marrow plasma cells in the WT–BA.2–BA.2 and WT–XBB–XBB groups was significantly higher than that in the WT–BA.2 and WT–XBB groups, respectively (left panels in Figure 4D,E). This suggests that the leveraged vaccination may have induced the presence of long‐lived plasma cells with residency in the bone marrow. We then used enzyme‐linked immunospot (ELISPOT) assay to identify antigen‐specific BMPCs (right panels in Figure 4D,E), revealing a significantly higher number of antigen‐specific BMPCs in the leveraged vaccination groups. Taken together, these findings demonstrate that leveraged vaccination effectively activates long‐lasting humoral immunity, while weakening the effects of OAS.

## 3 Discussion

Ancestral (WT) SARS‐CoV‐2 vaccines have been widely administered globally, and many people in the world have been infected by ancestral SARS‐CoV‐2 [15]. This means that individuals previously exposed to WT SARS‐CoV‐2 antigen will experience the effects of OAS upon immunization with booster vaccines based on SARS‐CoV‐2 variants or subvariants. This calls for the development of novel booster vaccines or vaccination strategies that can weaken OAS effect and therefore enhancing bnAb response against SARS‐CoV‐2 variants or subvariants currently in circulation or those expected to evolve in the future.

In this study, we have addressed the effects of OAS upon subsequent vaccinations and developed a leveraged vaccination strategy that takes into account antigenic distance such that utilizing booster vaccines with longer antigenic distance, along with a double booster vaccination protocol (Figure 1A), weakens OAS, while improving bnAb responses against Omicron subvariants.

Our study reveals that BA.2–RBD and XBB–RBD are genetically similar antigens (similarity: 98%), but exhibit distinct antigenic distances from WT–RBD (1.79 AU vs. 4.56 AU) (Figure 3D), thus making different contributions to OAS. The genetic distance provides essential evolutionary information about the variants of the fast‐evolving RNA viruses such as influenza virus and SARS‐CoV‐2. It is a critical metric for identifying viral lineages with potential for vaccine development [16]. Meanwhile, the antigenic distance is commonly used to evaluate the antigenic closeness between circulating strains and the current vaccine strain [17].

In general, the genetic distance is well corrected with the antigenic distances. When a virus undergoes gradual evolution, there is often a correlation between genetic distance and antigenic distance [18]. However, when a sudden acceleration in the evolution of the SARS‐CoV‐2 RBD happened, the correlation between these two will be affected [19].

For example, the SARS‐CoV‐2 virus has evolved in humans from the WT to the Alpha, Beta, Gamma, and Delta variants. In the RBD of the spike protein, these variants exhibit only 1 to 3 amino acid mutations, such as T478K and L452R in Delta variant. In contrast, when SARS‐CoV‐2 evolved from the WT to the Omicron variant, it acquired more than 15 amino acid mutations [20].

The numerous mutations observed in the RBD of Omicron appear to serve two purposes: enhancing its binding affinity to mouse ACE2 [21] and increasing its resistance to neutralizing antibodies [22]. According to a study in 2022, the Omicron variant may have undergone a transmission cycle involving humans and other animal species [23]. Researchers speculate that the SARS‐CoV‐2 initially infected humans and, in some cases, spread to cats [24]. After evolving to some degree in cats, the virus is thought to have been transmitted to mice, where it further adapted and eventually developed into the current Omicron variant before spreading back to humans [23].

In the Figure 1C, the sequence alignment showed that the similarity between the WT and BA.2–RBD were 95%, and relatively, the similarity between the BA.2 and XBB–RBD were 98%, indicating a high degree of similarity in genetic information. However, the antigenic distance between the WT and BA.2 is 1.79 AU, and that between the BA.2 and XBB is 2.71 AU. In conclusion, the amino acid mutations at the antigenic site of the Omicron subvariants can change the antigenic distance, suggesting that the genetic and antigenic distances do not always correlate. Serum nAbs elicited by the BA.2–RBD booster vaccine are effective against BA.2 and Omicron subvariants with similar antigenic distances, such as BA.2.2 (2.17 AU) and BA.2.75 (2.31 AU), but ineffective against Omicron subvariants with longer antigenic distances, such as XBB (4.56 AU) and JN.1 (4.64 AU). Notably, however, the serum nAbs elicited by XBB‐RBD booster vaccine are effective against XBB (4.56 AU) and Omicron subvariants with similar antigenic distances, such as XBB.1.5 (4.75 AU) and JN.1 (4.64 AU), but even more effective against SARS‐CoV‐2 variants and subvariants with shorter antigenic distances, for example, Delta (0.43 AU), BA.2 (1.79 AU), BA.2.2 (2.17 AU), BA.2.75 (2.31 AU), BF.7 (3.97 AU), and BA.5 (4.04 AU) (Figure 3D). These results suggest that the antigenic distance of booster vaccines is a key factor of this leveraged vaccination strategy and a determinant of bnAb response. In short, this means that we should select an antigen with antigenic distance as long as possible for the development of booster vaccines.

Another key factor of leveraged vaccination is the efficacy of double boosting vaccination. For example, double booster vaccination with XBB–RBD (WT–XBB–XBB) results in significantly increased titer of serum nAbs against Omicron subvariants, which is about 6‐ to 39‐fold higher than that in mice receiving single booster vaccination (WT–XBB) (Figure S4). However, since bnAb response elicited by WT–XBB–XBB is already strong enough to inhibit infection of the currently predominant Omicron subvariants, we are suggesting WT–XBB–XBB vaccination for vaccinees whose nAb titer has been reduced to a very low level.

In our study, we observed that immunization through a leveraged strategy effectively induced the production of bnAbs against the Omicron subvariants, without significantly enhancing neutralization of the WT (D614G) strain after the second booster dose (Figure S4). Yisimayi and colleagues [19] observed that in mice preimmunized with two doses of an inactivated vaccine, the neutralizing titer against the WT increased from 6784 to 11,196 following double booster immunizations with the XBB spike protein.

This indicated that the OAS effect was not fully reversed, but robust variant‐specific responses were improved after subsequent Omicron exposures as well. One possible explanation is that the two doses of the inactivated vaccine induced a stronger neutralizing titer against the WT strain [6], thereby exacerbating the OAS response. Another potential explanation is that differences in the immunization format [6] and antigen type [25] could influence the immune imprinting response, as both factors might shape the nature of this response. Notably, according to the reports, some other strategies can also only boost variant‐specific responses, and are unlikely to completely eliminate the dominance of the immune response toward the prime exposure. Other strategies such as extending the booster interval up to 3 months could significantly enhances NT_50_ values against all corresponding booster variants [19]. And the use of adjuvant‐enhanced booster immunizations [26] or excess of antigens [27] have also been shown to weaken the OAS effect.

Although the United States Food and Drug Administration (US FDA) has approved and authorized the updated mRNA COVID‐19 vaccines corresponding to the JN.1 and KP.2 subvariants, most other countries, including China, do not have such approved booster vaccines for use. However, several XBB‐based booster vaccines have been approved for application [28]. Our study has shown that double booster vaccination with XBB–RBD (WT–XBB–XBB) can induce strong bnAb response against JN.1 (Figure 4) since XBB–RBD and JN.1–RBD have similar antigenic distance (4.56 AU vs. 4.64 AU) (Figure 3D). Indeed, the subunit XBB booster vaccine (WSK‐V102C) containing RBD of the XBB.1.5 approved by the China National Medical Products Administration (NMPA) in June 2023 could induce robust nAb response against Omicron subvariants, including JN.1 [28]. The monovalent XBB.1.5 mRNA booster vaccine that received approval from the US FDA in September 2023 can also elicit robust nAb response against Omicron subvariants XBB and JN.1 [8]. Therefore, we would suggest using the XBB booster vaccine for double immunization when and where JN.1 or KP.2 booster vaccine is unavailable to combat JN.1 and future emerging Omicron subvariants if their antigenic distance similar to that of XBB.

While this study demonstrated the alleviated OAS effect in mice, it still encounters several limitations that call for further consideration. First and foremost, given the differences in the V(D)J gene sequences between humans and mice [29], which lead to variations in B‐cell responses to antigens or vaccines, this strategy necessitates further validation through clinical trials. Another significant limitation of this strategy lies in the selection of appropriate antigens. These antigens can only be identified after determination of the antigenic distance for the emerged virulent strains. Therefore, future studies should incorporate techniques, such as deep mutational scanning [30], to predict potentially prevalent antigens and optimize the antigen selection process.

OAS has also been observed in other rapidly evolving RNA viruses with immune escape mechanisms, such as influenza [31] and dengue viruses [32]. In the case of influenza, preexposure to seasonal flu strains may contribute to OAS, potentially diminishing the efficacy of pandemic or multivalent flu vaccines, such as observed in the study evaluating the BA.5 bivalent booster vaccine [33]. Therefore, our leveraged vaccination strategy can also be applied for attenuation of OAS caused by other rapidly evolving RNA viruses, as well as the rational design and development of booster vaccines against these rapidly evolving RNA viruses in current circulation or those expected to emerge in the future.

## 4 Materials and Methods

### 4.1 Cell Lines

Human embryonic kidney 293T (HEK293T) cells were obtained from the American Type Culture Collection, and human hepatoma Huh‐7 cells were from the Cell Bank of the Chinese Academy of Sciences (Shanghai, China). Both were cultured in Dulbecco's modified Eagle's medium (DMEM) supplemented with 10% fetal bovine sera (FBS) in a 37°C, 5% CO_2_ incubator. Expi 293F cells were cultured in OPM‐293‐CD05 medium (OPM Biosciences; Cat: 81075‐001).

### 4.2 Recombinant Protein Expression

All recombinant proteins were expressed as previously described [34]. In brief, variant‐specific RBD plasmids were transfected into Expi 293F cells separately using EZ‐Trans transfection reagent (Life‐lab Biotech; Cat: AC04L092). Cell supernatants were collected 6 days after transfection. RBDs were purified using Ni NTA Sepharose (Smart‐Lifesciences; Cat: SA004100) according to the manufacturer's instructions.

### 4.3 mRNA Formulation

The mRNA was transcribed and capped in vitro, using T7 RNA polymerase‐mediated transcription from linearized DNA templates, and the LNP formulations were prepared using the same procedure as previously described. Briefly, the lipid mixture was hedged with mRNA dissolved in 20 mM citrate buffer through a T‐mixer chip. Formulations were then diluted with phosphate buffered saline (PBS) (pH7.4) through a tangential flow filtration membrane with 100 kDa molecular weight cutoffs (Sartorius Stedim Biotech; Cat: WA10005DIS12LL), finally filtered with a 0.22 µm membrane, and stored at 2–8°C until use.

### 4.4 LNP Characterization

Dimensional measurements were made on a Malvern Zetasizer Nano‐ZS (Malvern) using DLS. A red laser (*λ* = 632.8 nm) was used with a backscatter angle of 173° for the scattered light. After analyzing the results using software (Zetasizer V7.13), an autocorrelation function was obtained [35]. For analysis of mRNA–LNP morphology, we used transmission electron microscopy [36]. A sample (3 µL) was deposited on a holey carbon grid that was glow‐discharged (Quantifoil; Au300 1.2/1.3) and vitrificated using a Vitrobot Mark IV instrument (Thermo Fisher Scientific). Cryo‐EM imaging was conducted on a Glacios Cryo Transmission Electron Microscope (Thermo Fisher Scientific) operated at 200 kV accelerating voltage.

### 4.5 mRNA Transfection and Expression Validation

In vitro expression was validated as previously reported [37]. HEK293T cells were seeded 24 h before transfection. Generally, 2 µg of mRNA were diluted in 100 µL Opti‐MEM (Thermo Fisher Scientific; Cat: 31985070) and mixed with Lipofectamine 3000 Transfection Reagent (Thermo Fisher Scientific; Cat: L3000015) before adding to cells. After 48 h, the cells were fixed with paraformaldehyde and 0.1% Triton X. After blocking with 3% BSA, cells were incubated with rabbit anti‐SARS‐CoV‐2 RBD antibody (1:2000; Sino‐Biological; Cat: 40592‐T62) for 30 min at 37°C. After washing the cells with PBS five times, goat anti‐rabbit IgG cross‐adsorbed secondary antibody, Alexa Flour 488 antibody (Thermo Fisher Scientific; Cat: A‐11008) was added to the cells to detect expressed RBD protein. Immunological histopathology was employed for in vivo visualization of the expression. Draining lymph nodes were collected 12 h after immunization. SARS‐CoV‐2 RBD rabbit polyclonal antibodies and donkey anti‐Rabbit IgG (H+L) cross‐adsorbed secondary antibody, HRP (Thermo Fisher Scientific; Cat: 31458) were used for detection.

### 4.6 Animal Information and Vaccination

Pathogen‐free, 8 weeks old female BALB/c mice were purchased from Beijing Vital River Laboratory Animal Technology Co., Ltd., and housed in the SPF‐grade barrier facility with a condition of 22 ± 1°C temperature, 50 ± 10% humidity, and 12:12 light/dark cycle. Operation and transportation of mice animals were carried out in strict accordance with the guidelines set by the Chinese Regulations of Laboratory Animals and Laboratory Animal Requirements of Environment and Housing Facilities. During the experiment, the mice were randomly assigned to groups, and the protocols for immunization of mice with placebo or the related RBD mRNA vaccines were shown in Figures 2, 3, 4A, and S2A. The vaccination timeline, antigen amount, and animal number for each group were described in the figure legends.

### 4.7 Pseudovirus Neutralization Assay

Pseudovirus neutralization assay was performed as previously described [38]. In brief, mouse sera were inactivated at 56°C for 30 min. Huh‐7 cells at a density of 7000 were seeded in wells of a 96‐well plate and incubated for 24 h. Mouse sera were threefold serially diluted in DMEM and incubated with the same volume of pseudovirus at 37°C for 45 min. Then, the mixtures were transferred into the wells and incubated in a 5% CO_2_ environment at 37°C for 12 h. Subsequently, fresh DMEM containing 5% FBS was added to replace the supernatant. After 48 h incubation, 1× firefly luciferase assay lysis buffer (Promega; Cat: E1531) was added to cells for 30 min. Finally, the cell lysate was transferred into a flat opaque 96‐well half‐area plate, and the luciferase value was measured using the luciferase assay system (Promega; Cat: E1500) with the SpectraMax Mini microplate reader (Molecular Devices).

### 4.8 Focus Reduction Assay for Testing the Neutralizing Activity of Antisera against Authentic BA.5.2 Infection

Vero‐E6 cells/well (1 × 10^4^) were seeded in wells of a 96‐well plate. Diluted sera from immunized mice were first mixed with authentic Omicron BA.5.2 for 30 min. Mixtures of virus and serum at series of dilution were added into the Vero‐E6 cells. After incubation for 48 h, cells were used to perform the focus reduction assay via immunofluorescence. Briefly, cells were treated with paraformaldehyde and 0.1% Triton X‐100. After blocking with 3% BSA, cells were incubated with mouse anti‐SARS‐CoV‐2 N antibody (1:2000; Sino Biological; Cat: 40143‐MM05) for 30 min at 37°C. After washing the cells with PBS five times, donkey anti‐mouse IgG (H+L) highly cross‐adsorbed secondary antibody, Alexa Fluor 488 (Thermo Fisher Scientific; Cat: A‐21202) was added to the cells and fluorescence focuses were accounted with a fluorescence microscope. The focus reduction and NT_50_ were calculated as previously described [39].

### 4.9 Flow Cytometry

Bone marrow cells were collected and smashed with a 100‐µm strainer to make a single cell suspension [40]. Cells were blocked with Trustain FcX anti‐mouse CD16/32 antibodies (BioLegend; Cat: 101320), followed by staining for viability with the zombie aqua fixable viability kit (BioLegend; Cat: 423102) for 15 min at room temperature in FACS staining buffer (Thermo Fisher Scientific; Cat: 00‐4222‐26). Fluorochrome‐conjugated antibodies in FACS staining buffer were FITC‐anti‐mouse/human CD45R/B220 antibody (BioLegend; Cat: 103205), PE‐anti‐mouse/human CD44 Antibody (BioLegend; Cat: 103007) and APC‐anti‐mouse CD138 (Syndecan‐1) antibody (BioLegend; Cat: 142505). Surface staining was carried out for 45 min at room temperature. Data were acquired in the BD LSRFortessa Cell Analyzer and analyzed using FlowJo analysis software.

### 4.10 ELISPOT Assay

To detect humoral responses, antigen‐specific antibody secreting cells based on ELISPOT assays were performed [41]. According to the ELISpot Flex: Mouse IgG ALP manufacturer's instructions (Mabtech; Cat: 3825‐2A), a 96‐well PVDF membrane‐bottomed plate was used in these experiments. After activation of the membrane, 50 µL of diluted antigens were added into each well of the plate and incubated overnight at 4°C. 1 × 10^6^ mouse bone marrow cells were added into each well of the plate and then incubated at 37°C for 36 h with 5% CO_2_. The cells were removed, and the plates were washed five times with PBS. Hundred microliters of diluted biotinylated detection antibody was added into each well of the plate and incubated for 2 h. Plates were washed with 1% BSA PBS five times, and then 50 µL diluted streptavidin‐conjugated alkaline phosphatase was added into each well and incubated for 1 h at RT. After washing five times, 100 µL AEC (3‐amino‐9‐ethylcarbazole) HRP substrate were added to each well for 10 min, and the reaction was stopped by washing under tap water. Images were captured using an automatic ImmunoSpot analyzer.

### 4.11 Antigenic Cartography

Antigenic distances between serum samples and WT, along with other SARS‐CoV‐2 variants, were calculated by integrating the GM NT_50_ values of immunization group serum samples using a published antigenic cartography method [42]. Visualizations were created with the Racmacs package (version 1.1.4, https://acorg.github.io/Racmacs/) within R software version 4.0.3. Multidimensional scaling was then performed on the distance table to generate the map. Optimization was set to 10,000 steps.

### 4.12 Phylogenetic Analysis

The phylogenetic analysis was performed as previously studies described [8]. RBD protein sequences alignment was conducted using the MEGA 11 program (version 11.0.13), and we conducted a neighbor joining phylogenetic analysis using the defaulted settings and visualized the results with the MEGA 11 program [43] (version 11.0.13). The phylogenetic tree was further modified in the iTOL [44] tool (https://itol.embl.de/itol.cgi).

### 4.13 Quantification and Statistical Analysis

All statistical analyses were performed using GraphPad Prism 9. Results are presented as GM ± geometric SD. Paired *t*‐test and one‐way ANOVA with Tukey's multiple comparison tests were used to evaluate statistical significance. Results with *p* < 0.5 were considered statistically significant. Statistical significance labels: *p < 0.05; **p < 0.01; ***p < 0.001.

## Author Contributions

S.J., L.L., Q.W., and B.Y. conceptualized the study. G.Z., K.J., Y.W., and X.L. conducted experiments for the mRNA vaccine synthesis, validation and characterization. G.Z., Y.W., W.X., R.X., L.X., Z.L., Jie Zhou, and Jianghao Zhou performed the mouse vaccination, virus neutralization, flow cytometry, and ELISPOT assays. G.Z., Q.W., X.W., and Y.S. carried out the data and statistical analysis and antigenic cartography. G.Z., Q.W., L.L., and S.J. wrote the manuscript. All authors have read and approved the final manuscript before submission.

## Ethics Statement

Animal studies were strictly followed and approved by the Institutional Laboratory Animal Care and Use Committee at Fudan University, Shanghai, China (Approval number: 20230629‐001).

## Conflicts of Interest

In this manuscript, the mRNA vaccines utilized are subject to patents held by Suzhou Abogen Biosciences Co., Ltd. The authors Kai Ji and Xishan Lu are affiliated with this company as employees. Nevertheless, they have no potential relevant financial or nonfinancial interests to disclose. The other authors have no conflicts of interest to declare.

## Supporting information



Supporting information

## Data Availability

All data used for the current study are available from the corresponding author upon reasonable request.
