# Supplementary material for: Leveraged Vaccination to Alleviate Original Antigenic Sin for Enhancing Broad‐Neutralizing Antibody Response against SARS‐CoV‐2 Omicron Subvariants
Source: MedComm (2020). 2025 Jul 7;6(7):e70273. doi: 10.1002/mco2.70273 (PMC12231049; doi:10.1002/mco2.70273)
Supplement: Supplementary file 1 — Supporting information [file MCO2-6-e70273-s001.docx]

**Supplemental Information**

**Leveraged vaccination strategy to alleviate original antigenic sin for enhancing broad-neutralizing antibody response against SARS-CoV-2 Omicron subvariants**

Guangxu Zhang ^1,#^, Qian Wang ^1,#^, Kai Ji ^2,#^, Yuanzhou Wang ^1,#^, Wei Xu ^1^, Jie Zhou ^1^, Zezhong Liu ^3^, Ruixue Xiu ^1^, Lixiao Xing ^1^, Jianghao Zhou ^1^, Yuren Shi ^1^, Xishan Lu ^2^, Xuanyi Wang ^1^, Bo Ying ^2,*^, Lu Lu ^1,*^, Shibo Jiang ^1,*^

^*^ **Correspondence**: shibojiang@fudan.edu.cn (S. Jiang), lul@fudan.edu.cn (L. Lu), and bo.ying@abogenbio.com (B. Ying).

**This Word document includes Figures S1 – S5.**

**Supplementary Figure S1**

**
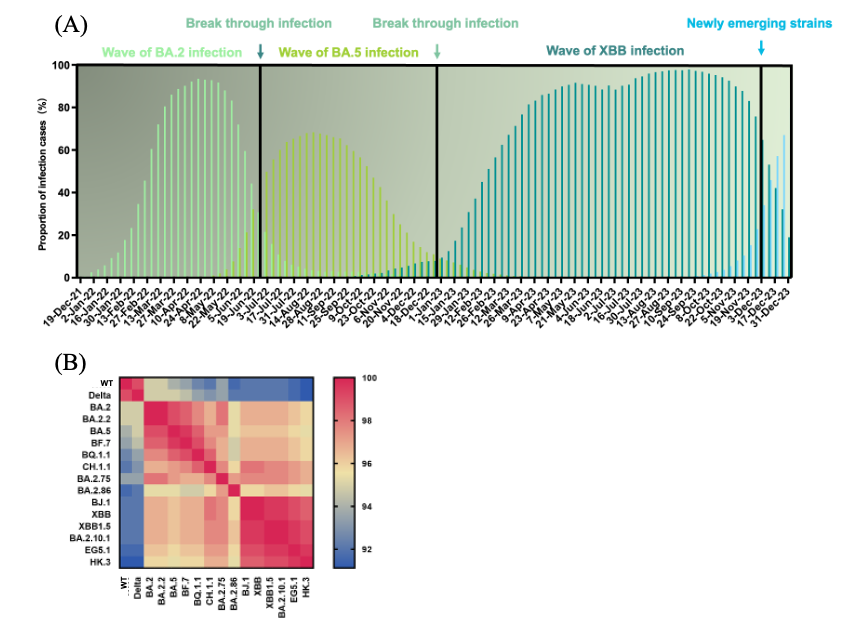
**

**FIGURE S1** BA.2- and XBB-RBD are genetically distinct based on sequence alignment.(A) Distribution of Omicron subvariants that have emerged worldwide, starting in early October of 2022 through the beginning of January 2024. Data were collected from GISAID and plotted using GraphPad Prism. (B) Sequence similarity analysis for RBDs of SARS-CoV-2 and its variants and subvariants.

**Supplementary Figure S2**


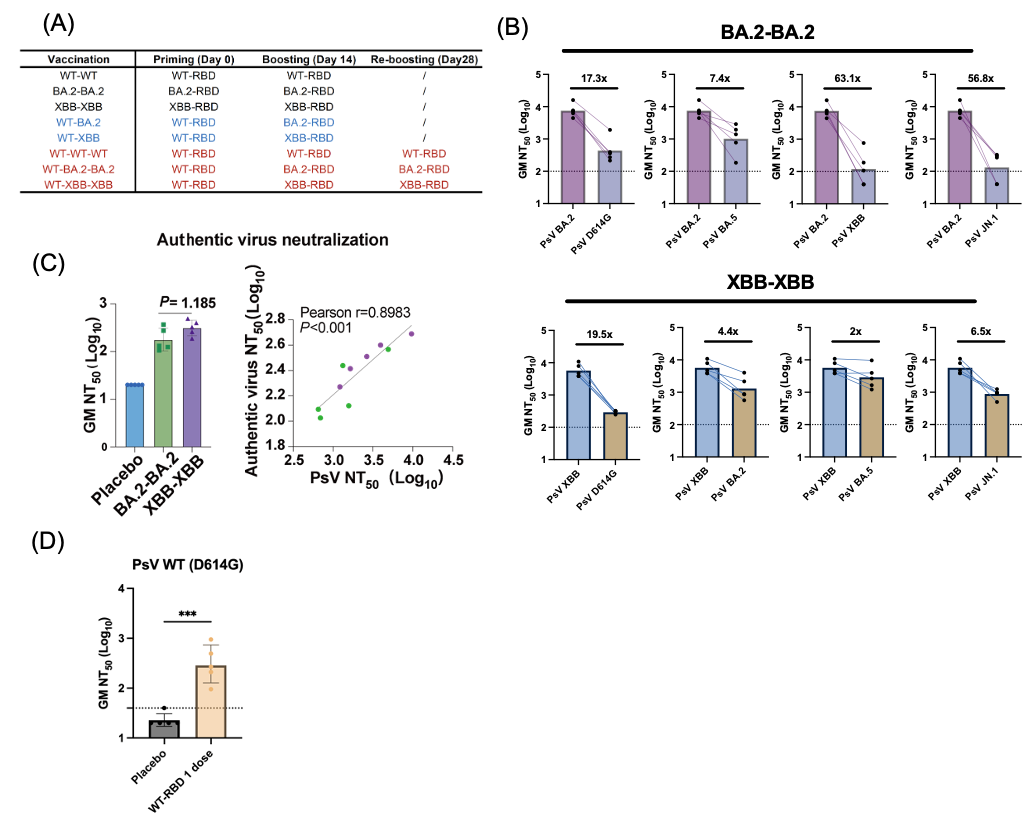


**FIGURE S2** Serum neutralization landscapes before and after leveraged vaccination. (A) Vaccination procedures in this study. (B) Fold changes in GM NT_50_s of antibodies in sera of mice collected at day 14 after immunization with homologous booster vaccines against PsVs of WT-D614G, BA.2. BA.5, XBB, and JN.1 compared with those against PsV of BA.2 (upper panel) or XBB (lower panel). Statistical analyses were performed using paired T test for the decrease of GM NT_50_ in other variants compared to BA.2 for the authentic neutralization antibody titers. (C) Focus reduction assay was used for assessment of GM NT_50_ in sera of mice vaccinated with BA.2-BA.2, XBB-XBB, or placebo against BA.5.2 authentic virus (left panel). Correlation between pseudotyped and authentic BA.5 neutralization titers in sera collected from Balb/c mice on day 28 post prime immunization (right panel). Statistical analyses were performed using one-way ANOVA for comparison of the neutralization antibody titers. (D) Evaluation of the neutralization base line reference of mouse sera collected after the first prime immunization with WT-RBD against the WT (D614G) pseudovirus neutralization titer. Statistical analyses were determined using student T test for comparison of the neutralization antibody titers.

**Supplementary Figure S3**


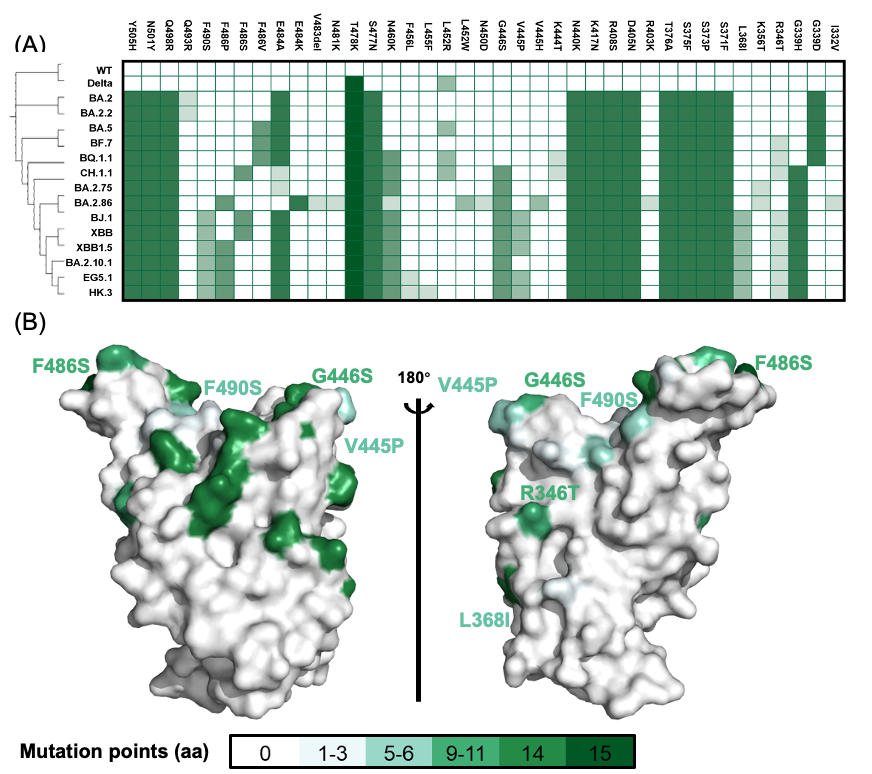


**FIGURE S3** Mutations in RBD of SARS-CoV-2 and its variants and subvariants. (A) Accumulation of mutations in RBDs based on sequence alignment. (B) Mutation hot spots on the RBD (PDB ID: 7KMG). The gradient color stands for the mutation frequency of the amino acid points in RBDs of the strains in (A), and the color scales was labeled below.

**Supplementary Figure S4**

**
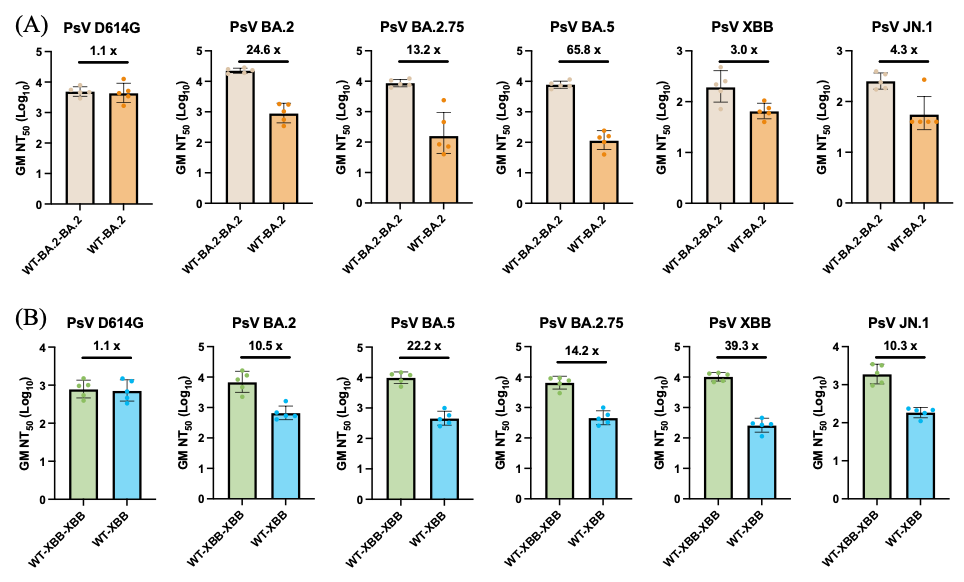
**

**FIGURE S4** Relative improvement in neutralization titers of WT-BA.2-BA.2 and WT-XBB-XBB against D614G, BA.2, BA.5, BA.2.75, XBB, and JN.1 compared to WT-BA.2 and WT-XBB. (A) Fold changes in GM NT_50_s of WT-BA.2-BA.2 against D614G, BA.2, BA.5, BA.2.75, XBB, and JN.1 PsVs compared to those in WT-BA.2 for sera collected at day 14 after final boosting. (B) Fold changes in GM NT_50_s of WT-XBB-XBB against D614G, BA.2, BA.5, BA.2.75, XBB, and JN.1 PsVs compared to those in WT-XBB for sera collected at day 14 after final boosting.

**Supplementary Figure S5**


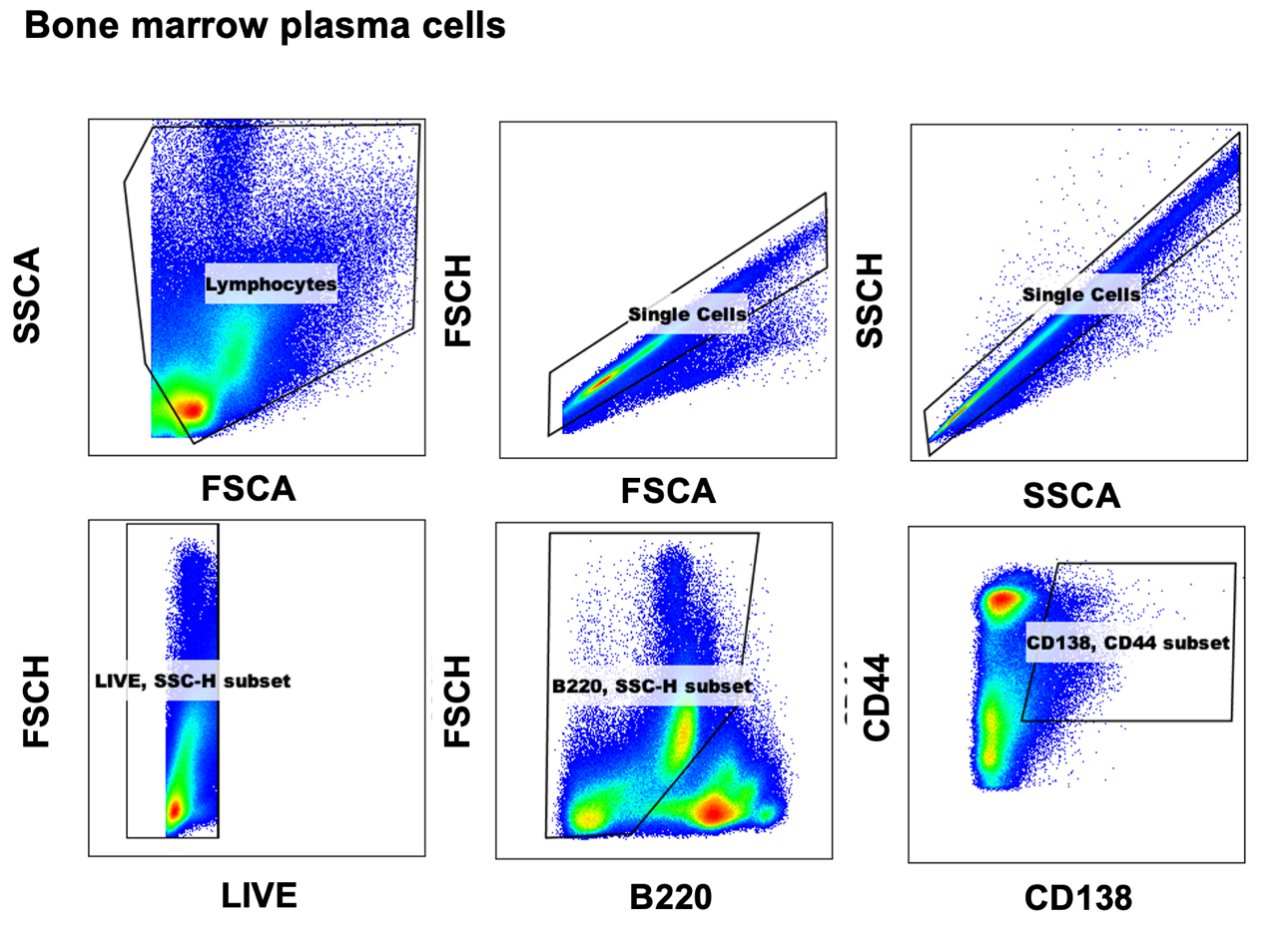


**FIFURE S5** Gating strategies for bone marrow plasma cells. Flow cytometry for the bone marrow cells collected 42 days (single boosting) and 56 days (double boosting) after the first immunization and the BMPCs were marked as CD138^+^ and CD44^+^.

**Supplementary Figure S6**


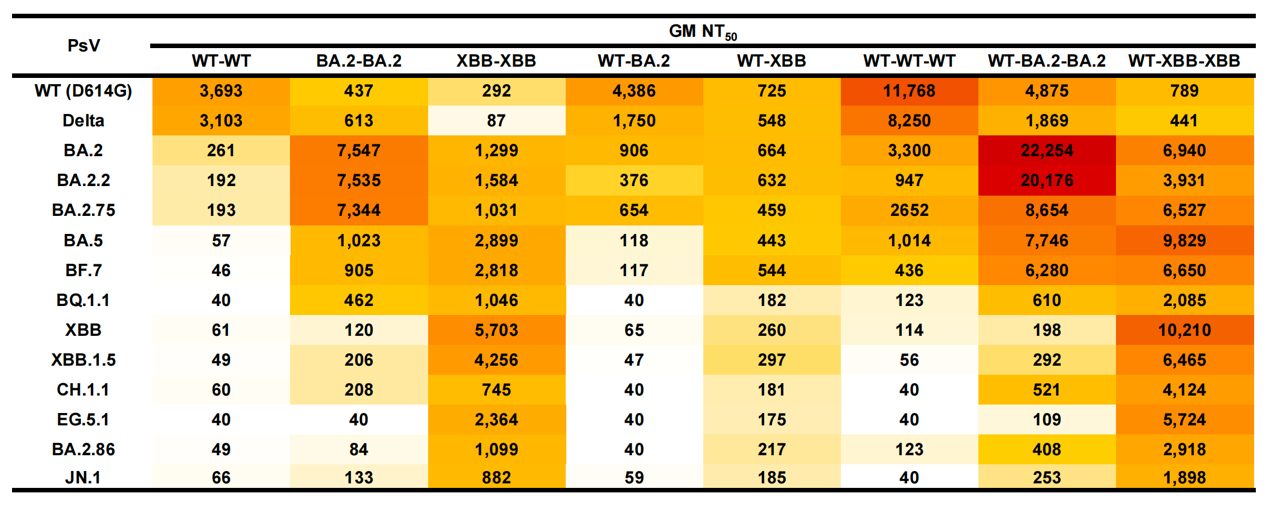


**FIFURE S6** Neutralization titers against 14 types of SARS-CoV-2 variants. a total of 8 groups of mouse antisera GM NT_50_ were summarized in the table. The values were labeled in Red (high value) - yellow (medium value) - white (low value) gradient scales. The limits of the detection in this study is 1:50 in dilution, and the individual NT_50_s under this limits are considered as 40.
